# Landscape Heterogeneity Drives Plant Assemblage Dynamics and Invasibility of Semi-Natural Grasslands Under the Long-Term Invasion of *Ageratina adenophora*

**DOI:** 10.3390/plants15060862

**Published:** 2026-03-11

**Authors:** Longyuan Zhao, Lirong Guan, Qianmei Zou, Lu Xu, Yang Wang, Ninghui Pan, Sitong Liu, Shaorong Wu, Dexi Wu, Yong Xie

**Affiliations:** 1 College of Plant Protection, Yunnan Agricultural University, Kunming 650201, China; longyuan709@outlook.com (L.Z.); qianmei_zou@hotmail.com (Q.Z.); 13759170135@163.com (L.X.); wangyang626@sina.com (Y.W.); 15398535242@163.com (N.P.); sitongl0408@163.com (S.L.); 2 College of Chemical Engineering, Yunnan Open University, Kunming 650500, China; glr080125@163.com; 3 State Key Laboratory for Conservation and Utilization of Bio-Resources in Yunnan, Yunnan Agricultural University, Kunming 650201, China; 4 Agricultural Science and Technology Service Center of Huaning County, Yuxi 652899, China

**Keywords:** *Ageratina adenophora*, important value, indigenous plant, interspecific competition, replacement management, semi-natural grasslands

## Abstract

Grassland degradation is a critical ecological problem worldwide that threatens ecosystem integrity and functional services. Although previous studies have documented the drivers of climate change, overgrazing, and anthropogenic perturbation, research concerning the impact of invasive alien plants on grassland ecosystems remains limited. The present study, integrating pairwise field investigation of *Ageratina adenophora* invasion and non-invasion plots across heterogeneous grassland types (tropical grasslands [TG]; tropical shrub-grasslands [TS]; warm-temperate grasslands [WG]; and warm-temperate shrub-grasslands [WS]) and *A. adenophora* indigenous plants phytotoxicity bioassay, aims to assess the invasibility and resilience of heterogeneous grassland landscapes to *A. adenophora* invasion. The field investigation demonstrated the greater vulnerability of TG and TS to *A. adenophora* invasion, whereas WG and WS possessed higher resilience. In addition, regression analysis revealed significant reductions of the Shannon–Wiener index and the Pielou index as the *A. adenophora’s* important value reached the threshold 0.36. Bioassay showed that *A. adenophora* aqueous extracts inhibit seed germination and seedling growth of recipient plants, with *Saccharum arundinaceum* exhibiting the highest tolerance to *A. adenophora* stress. In summary, our findings not only highlight the flora communities’ dynamics and invasibility of diverse grasslands driven by *A. adenophora* invasion in subtropical regions but also verify *S. arundinaceum’s* potential for *A. adenophora* replacement management.

## 1. Introduction

Grasslands cover approximately 26% of the global terrestrial area, playing a crucial role in ecosystem services, including maintaining biodiversity, carbon sequestration, climate regulation, and biogeochemical cycles [1,2,3,4]. In China, grasslands occupy approximately 40% of the national land area, equivalent to more than 263 million hectares [5]. Semi-natural grasslands serve as habitats for indigenous herbaceous plants, and are characterized as either unimproved or grazed herd pastures that account for 80.59% of the national grassland area, and are predominantly distributed across Tibet, Inner Mongolia, Xinjiang, Qinghai, Gansu, Sichuan, and Yunnan Province [6]. It has been widely recognized that the functional services provided by grassland ecological barriers play a positive role in ecological conservation and defense against alien plant invasion [7,8,9]. However, both theoretical understanding and practical implementation remain insufficient, constraining the effectiveness of related projects and policies [10].

Yunnan Province, one of the world’s biodiversity hot spots, located in southwestern China and bordering South and Southeast Asian countries, contains semi-natural grasslands that not only provide pasture and habitat for livestock and diverse wildlife but also serve as ecological barriers against alien invasive organisms migrating from Vietnam, Laos, and Myanmar [11,12]. However, with the rapid growth of China-ASEAN international travel and merchandise importation, the potential risk of invasive alien pests unintentionally introduced into Yunnan and inland increased.

*Ageratina adenophora*, one of the most aggressive alien Asteraceae species, has raised serious concern among both the public and the academic community worldwide [13,14,15]. In China, *A. adenophora* was first introduced from Myanmar to Lincang, a prefecture of Yunnan Province, in the 1940s, and rapidly spread inland along the southwestern monsoon [16]. The impact of *A. adenophora* invasion on plant communities exhibits a complex process and uncertain consequences. Generally, following the alien invasion exacerbation, it leads to a decline in the diversity of native plant species [17,18]. However, in certain cases, the invasion of *A. adenophora* can also exert a positive effect by increasing the species richness within the native range [19,20]. Consequently, the question arose: besides geographical proximity and climatic similarity, how has the landscape and invasion stage driven local flora composition dynamics? Thus, considering the contradictory perspectives and the insufficient studies focusing on semi-natural grasslands, a multidimensional study addressing the long-term invasion impact of *A. adenophora* on plant species diversity of grassland ecosystems is required.

It is widely recognized that maintaining the level of species diversity is crucial for sustaining ecosystem stability and integrity [21,22,23]. In addition, species diversity represents a fundamental characteristic of grasslands and an important indicator for assessing the functions of grassland ecosystems [24,25,26]. Therefore, assessing plant species diversity in grasslands facilitates a comprehensive understanding of floral community dynamics and structural attributes [27,28]. Although numerous government-initiated programs and related research have recently achieved considerable progress in managing grassland alien species, studies focusing on diverse tropic/subtropic semi-natural grasslands addressing diversity loss in long-term invasion scenarios, especially the species assemblage dynamic on the local scale, remain comparatively limited. In addition, the ecological response of grasslands to *A. adenophora* invasion, represented by invasibility and resilience of different grassland types, is still unknown. Hence, to address this research gap concerning the long-term impact of *A. adenophora* on grasslands, the present study, conducted in the semi-natural grasslands of Chengjiang County in central Yunnan, integrates field surveys and laboratory bioassays to: (1) clarify the invasibility of diverse grassland landscapes under long-term *A. adenophora* invasion; (2) determine the phytotoxic effects of *A. adenophora* on coexisting indigenous plant species; and (3) assess the feasibility and propose biological solutions using indigenous plant replacement for *A. adenophora* management.

## 2. Materials and Methods

### 2.1. Study Area

Chengjiang County, located in central Yunnan Province, China, at geographic coordinates 24° 29′–24° 55′ N and 102° 47′–103° 04′ E, has a mountainous administrative area of 773 km^2^. It is renowned for Fuxian Lake, the largest national deep freshwater lake, which plays an important role in local ecological preservation and regional climate regulation.

**Figure 1:**
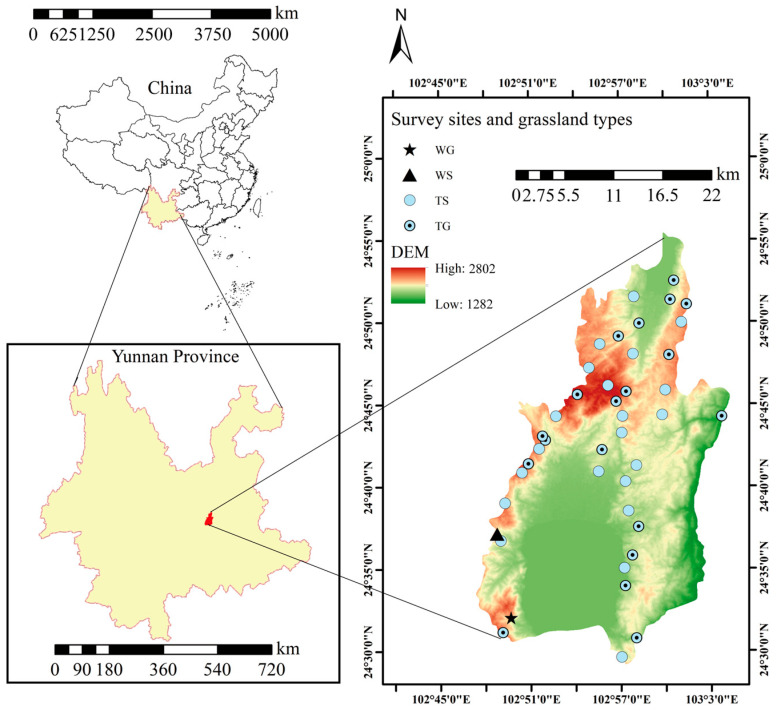
Overview of study area in grasslands of Chengjiang County. Warm-temperate grasslands (WG); warm-temperate shrub-grasslands (WS); tropical grasslands (TG); tropical shrub-grasslands (TS).

**Figure 2:**
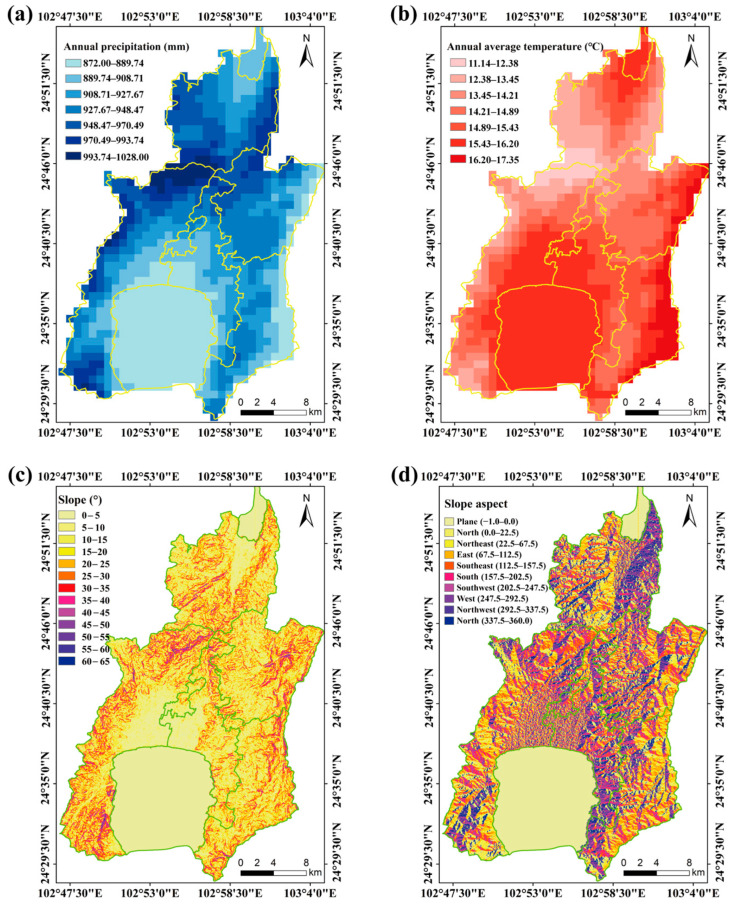
Topographical and climatic features of Chengjiang County, Yunnan Province. (**a**), Annual precipitation; (**b**), Annual average temperature; (**c**), Slope; (**d**), Slope aspect. The data cited from the World Climate Database (https://www.worldclim.org/) and Geospatial Data Cloud (http://www.gscloud.cn/).

The county includes 3600 hm^2^ of grassland, encompassing four grassland types: tropical grasslands (TG), tropical shrub–grasslands (TS), warm-temperate grasslands (WG), and warm-temperate shrub–grasslands (WS) (Figure 1; Table S1). The region experiences a monsoon climate characteristic of the central subtropical plateau, with an annual precipitation ranging from 872 to 1028 mm (Figure 2a) and an annual temperature range of 11.14–17.35 °C (Figure 2b). It is a typical hilly area in the central Yunnan Plateau, with a maximum slope of 65° (Figure 2c) and predominantly east–northwest slope orientation (Figure 2d). The highest elevation, 2802 m, is found at Mount Liangwangshan, while the lowest elevation, 1282 m, occurs at the confluence of the Nanpanjiang and Haikou Rivers.

### 2.2. Investigation Methods and Data Collection

#### 2.2.1. Plot Design and Investigation

The survey was conducted in the Chengjiang grasslands between June and September 2023. Based on the grassland types (TG, TS, WG, and WS) and corresponding acreage, pairwise plots (invasion plot and corresponding non-invasion plot) were strictly selected with identical grassland types and matched ecological characteristics (elevation, slope, slope aspect, soil type, and grazing pressure) to ensure ecological equivalence except for the presence of *A. adenophora*. The total number of surveyed plots was 56 (28 invasion plots and 28 non-invasion plots), with the specific number for each grassland type as follows: TG (10 invasion, 10 non-invasion), TS (14 invasion, 14 non-invasion), WG (2 invasion, 2 non-invasion), WS (2 invasion, 2 non-invasion). Specifically, in each 0.5 hm^2^ plot, the simple random sampling method was applied to establish five small quadrats (1 × 1 m) [29]. The invasion plot area covered approximately 0.5% of each grassland type based on the principle of stratified random sampling, which ensures that the sampling range covers the main distribution area of *A. adenophora* in each grassland type and thus achieves maximum representativeness of the invasion status [29]. During the survey, the abundance, height, and coverage of plant species were documented, along with ecological indicators, longitude, latitude, and elevation. Plant height was measured using a scale with an accuracy of 1 mm, whereas abundance was visually assessed by counting the above–ground parts of individual plants.

#### 2.2.2. Meteorological and Geographic Data Sources

Climate variables were obtained from the World Climate Database (https://www.worldclim.org/), extracting two variables: annual mean temperature and annual precipitation. The Spatial Analyst tool in ArcGIS 10.8 was used for sampling and extracting climate data. Topographic variables were derived from the Geospatial Data Cloud (http://www.gscloud.cn/), where DEM data, slope, and aspect analyses were performed using the 3D Analyst tool in ArcGIS 10.8.

### 2.3. Effect of A. adenophora Aqueous Extract on Indigenous Plants’ Seedling Growth

Herbaceous species with high occurrence frequency found in both invasion and non–invasion plots were selected as recipient plants against *A. adenophora* extracts stress, including *Imperata cylindrica*, *Saccharum arundinaceum*, *Eragrostis ferruginea*, *Calamagrostis epigeios*, and *Rumex hastatus*. Plants of *A. adenophora* and seeds of the recipient plants were collected from the invasion plots. Fresh leaves of *A. adenophora* were shade-dried, ground, and sieved through a 40-mesh sieve. An aqueous extract was prepared by soaking 5 g of leaf tissue in 100 mL of distilled water for 48 h, followed by filtration through two layers of gauze to obtain a 50 g/L stock solution. Extract concentrations of 25, 14, and 7 g/L were prepared by gradient dilution (1:2, 1:3.5, 1:7) with distilled water based on the pre-experiment results and previous reports, while distilled water served as the control [30,31]. Each concentration was dissolved in agar at 10 g/L with heating. Then, 20 mL of the agar solution was poured into 9-cm-diameter petri dishes containing five layers of filter paper to create agar-based germination substrates. The seeds of herbaceous plants were surface–sterilized in 0.1% (*w*/*v*) NaClO solution for 10 min, rinsed thoroughly with distilled water three times, and air-dried. Fifty seeds of each herbaceous species were placed on filter paper for the germination assay, with five replicates per extract concentration (including the control), resulting in a total of 120 petri dishes (5 plant species × 4 concentrations × 6 replicates). The seeds were then cultivated in an intelligent artificial climate chamber (PRX–1200B, Ningbo Saifu Experimental Instrument, Ningbo, China) under 16 h of illumination, 8 h of darkness, at a temperature of 25 ± 1 °C, and relative humidity of 90% ± 0.8 (%RH). Germination counts were recorded daily for each treatment. On the 20th day of cultivation, 30 seedlings per treatment were randomly selected for root length and shoot height measurements. An additional 20 seedlings per treatment were harvested to determine fresh weight. Germination and seedling vigor were calculated based on the following formulas [32]:

(1) $$Germination\;energy\;\left(GE,\;\%\right)=\frac{N_{3}}{N_{total}}\times 100\%$$

(2) $$Germination\;rate\;\left(GR,\;\%\right)=\frac{N_{germ}}{N_{total}}\times 100\%$$

(3) $$Germination\;index\;\left(GI\right)=\sum \left(G_{t}\times D_{t}-1\right)$$

(4) Vigor index (VI)=W×GI

where *N_3_* is the number of seeds germinated on the 3rd day; *N_total_* is the total number of seeds tested; *N_germ_* is the cumulative number of germinated seeds; *G_t_* is the number of seeds germinated on t day; *D_t_* is the number of days to germination; *W* is the average fresh weight (g) of 20 seedlings.

### 2.4. Data Analysis

The kernel density estimation (KDE) analysis was conducted using ArcGIS 10.8 to clarify and visualize the spatial distribution and relative abundance of *A. adenophora* in grasslands. The variation in density was measured using an established distance decay function, which was employed to explore the distribution and changing characteristics of density hot spots within spatial regions [33]. The invasion intensity index and community invasibility index were calculated to assess the invasion intensity of *A. adenophora* and the degree of community invasibility [34].

(5) $$Relative\;abundance\;\left(P_{i}\right)=\frac{n_{i}}{N}\times 100\%$$

(6) $$Community\;invasibility\;index\;\left(CII\right)=1-\left(MaxP_{i}-P_{i}\right)$$

(7) $$Invasion\;intensity\;index\;\left(III\right)=P_{i}/MaxP_{i}$$

where *n_i_* is the number of individuals of species i in the plot; *N* is the total number of all species in the individual plot; *Pi* is the observed relative abundance of *A. adenophora* in one invaded plot; *MaxPi* is the maximum of the relative abundance of *A. adenophora* among all invaded plots.

The following formula is utilized for the importance value and alpha diversity calculation [35].

(8) $$Relative\;height\;\left(RH_{i}\right)=\frac{Mean\;height\;of\;species\;i}{Total\;height\;of\;all\;spcies}\times 100\%$$

(9) $$Relative\;density\;\left(RD_{i}\right)=\frac{number\;of\;individuals\;of\;species\;i}{Total\;number\;of\;individuals\;of\;all\;species}\times 100\%\;$$

(10) $$Relative\;coverage\;\left(RC_{i}\right)=\frac{Coverage\;of\;species\;i}{Total\;coverage\;of\;all\;species}\times 100\%$$

(11) $$Importance\;value\;\left(IV\right)=\frac{RH_{i}+RD_{i}+RC_{i}}{3}$$

(12) Richness index (S)=Species number in plot

(13) $$Shannon\;Wiener\;index\;\left(H^{\prime }\right)=-\sum_{1}^{s}\mathrm{Pi}\;\ln\;\mathrm{Pi}$$

(14) $$Pielou\;index\;\left(E\right)=\frac{-\sum_{1}^{s}\mathrm{Pi}\;\ln\;\mathrm{Pi}}{\ln\;S}$$

(15) $$Simpson\;index\;\left(D\right)=1-\sum_{i=1}^{s}Pi^{2}$$

The similarity index was utilized to compare differences in species abundance and community composition between paired invasion and non-invasion plots for each grassland type to validate the impact of *A. adenophora* invasion on community composition. The Jaccard similarity index was applied to examine differences between all plots, and species were quantified based on presence–absence data. β-diversity was also assessed using the Sorensen similarity index [36,37].

(16) $$Jaccard\;similarity\;index=\frac{c}{a+b+c}$$

(17) $$Sorenson\;similarity\;index=\frac{2c}{a+b+2c}$$

where *a* denotes the number of species recorded in invasion plots; *b* is the number of species in non-invasion plots; and *c* is the number of species common to both plots.

Microsoft Excel 2019 was used for data organization and for calculating Alpha and Beta diversity. IBM SPSS Statistics 26 was utilized to perform a one-way ANOVA test, with results expressed as mean values and standard errors, as well as Pearson and Spearman correlation analyses. Piecewise regression analysis (using IBM SPSS Statistics 26) was used to determine the threshold of *A. adenophora* important value (IV) at which the Alpha diversity index changes significantly, with the threshold point identified by the inflection point of the regression curve. Origin 2021 was employed for plotting figures, while ArcGIS 10.8 was employed for extracting data and mapping the climate and topography of the survey plots, as well as for kernel density estimation (KDE) analysis.

## 3. Results

### 3.1. KDE Analysis of the Local-Scale Geographic Spatial Pattern of A. adenophora

**Figure 3:**
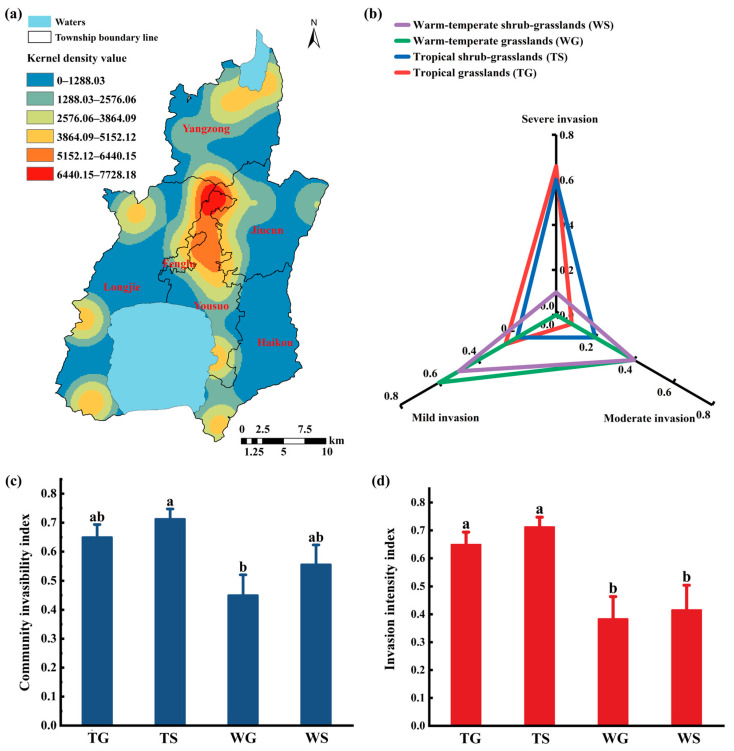
Community invasibility and invasion intensity analysis of *A. adenophora* in the grasslands of Chengjiang County. (**a**) Schematic kernel density estimation analysis of *A. adenophora* distribution in Chengjiang grasslands by using ArcGIS; (**b**) invasion degree of *A. adenophora* in different grassland types; (**c**) community invasibility index of different grassland types; (**d**) invasion intensity index of *A. adenophora* in different grassland types. Error bars in (**c**,**d**) represent mean ± standard error of 5 replications, different lowercase letters indicate significant differences at different grasslands (*p* < 0.01).

The KDE analysis revealed that *A. adenophora* was distributed in a mosaic and unevenly scattered pattern across six townships in the grasslands. Higher density values, ranging from 5152 to 7728, converged in the region spanning three townships, Yousuo, Jiucun, and Fenglu, which are located either in central municipal townships or rural–urban fringe areas. In contrast, the areas of Haikou, Longjie, and Yangzong, which are mainly rural and distant from downtown or major communication lines, exhibited comparatively lower density, with an approximate average kernel density value of 3560 (Figure 3a). This result confirmed that transportation convenience and anthropogenic disturbances exacerbated the propagation of *A. adenophora* through human-mediated seed dispersal [38].

### 3.2. Comparative Analysis of A. adenophora Invasion Degree in Different Grassland Types

A total of 28 invasive plots of *A. adenophora* were investigated. Statistically, the invasion plots were categorized as 27.14% mildly invasive, 18.57% moderately invasive, and 54.29% severely invasive plots. Among the different grassland types, TG (10 plots) and TS (14 plots) exhibited the highest proportions of severe invasion (66.00% and 60.00%, respectively). In contrast, WG and WS, with 2 plots each, were predominantly mildly invaded, at rates of 60.00% and 50.00%, respectively (Figure 3b). The results indicated that the invasion degree in both TG and TS was higher than that in WG and WS, demonstrating that TG and TS experienced greater damage caused by *A. adenophora* invasion.

### 3.3. The Community Invasibility Variation of Individual Grassland Types

The grassland community invasibility analysis revealed that the community invasibility index followed a descending order: TS > TG > WS > WG (*p* < 0.05) (Figure 3c). In contrast, although TS exhibited the highest average invasion intensity index, the differences among grasslands were not significant (Figure 3d). This finding indicated that, compared to WG and WS, both TS and TG were more vulnerable to *A. adenophora* invasion, possessing stronger community adaptability and invasion competitiveness.

### 3.4. Plant Community Comparison Between Invasion and Non-Invasion Grasslands

**Figure 4:**
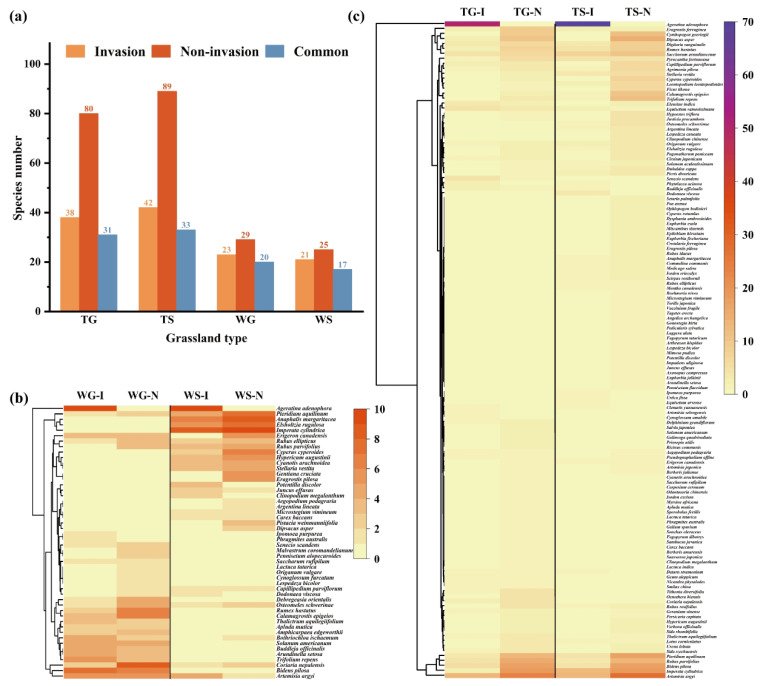
Plant species composition in different grassland communities. (**a**) Species number; (**b**) clustering analysis of plant species composition in WG and WS; (**c**) clustering analysis of plant species composition in TG and TS. (The upright scale represents plant species frequency in plots; I, invasion plots; N, Non-invasion plots).

Although both invasion (I) and non-invasion (N) plots share several plant species (common species), the statistics consistently demonstrated a reduction in plant species numbers in invasion plots across all grassland types (Figure 4a). TG and TS exhibited significantly higher overall species richness than WG and WS, but invasion plots of all grassland types demonstrated considerable species loss compared to non-invasion plots (TG 52.50% loss, TS 52.81% loss, WG 20.69% loss, and WS 16.00% loss). Cluster analysis showed that plant species can be divided into four complementary clades (Figure 4b,c). Specifically, for WT and WS, *A. adenophora* invasion significantly altered community composition, with both grassland types comprising more species in non-invasion plots than in the corresponding invasion plots (Table S3). Remarkably, a small number of species (e.g., *Phragmites australis*, *Ipomoea purpurea*) were only present in invasion plots, while a variety of native species (e.g., *Pennisetum alopecuroides*, *Malvastrum coromandelianum*) were exclusive to non-invasion plots, indicating that *A. adenophora* invasion squeezed native plants from habitats and distinctly altered community composition (Figure 4b).

TG and TS possess more shared species between invasion and non-invasion plots (31 vs. 33) compared to WG and WS (20 vs. 17), but still suffered severe loss of native exclusive species (TG and TS had 47 and 56 exclusive native species in non-invasion plots, respectively), indicating TG and TS possess a comparatively higher tolerance to *A. Adenophora* invasion (Figure 4c). The investigation results demonstrated distinct plant species compositions across different grassland types, reflecting the drastically diversified ecogeography in the survey area. Generally, TG and TS fostered a more diverse flora than WG and WS (Figure 4a). In all grassland types, over 20 plant species coexisted in both invasion and non-invasion plots.

### 3.5. Alpha Diversity

**Figure 5:**
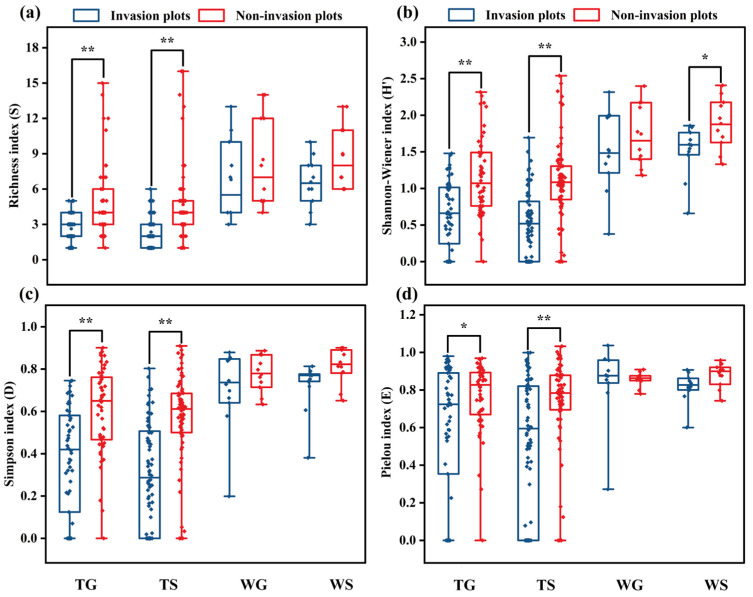
Alpha diversity comparison between grassland types. (**a**) Richness index; (**b**) Shannon–Wiener index; (**c**) Simpson index; (**d**) Pielou index. * indicates significant difference (*p* < 0.05); ** exhibits highly significant difference (*p* < 0.01).

Alpha diversity analysis showed that the Richness index (S) was higher in non-invasion than in invasion plots across all grassland types. Specifically, for TG and TS, there were highly significant differences (*p* < 0.01) between the pairwise plots (Figure 5a). Likewise, regarding the Shannon–Wiener index (H′), all grassland types exhibited a trend similar to the Richness index between non-invasion and invasion plots for WS; there was a significant difference (*p* < 0.05) between them (Figure 5b). The Simpson index (D) was higher in non-invasion plots than in invasion ones across all grassland types, indicating that *A. adenophora* invasion decreased species diversity within individual grassland communities. Except for WG and WS, there were highly significant differences (*p* < 0.01) between invasion and non-invasion plots in TG and TS (Figure 5c). The Pielou index (E) of non-invasion plots was higher than that of invasion plots, except for WG. Meanwhile, it exhibited a significant difference (*p* < 0.05) between invasion and non-invasion plots in TG, and for TS, there was a highly significant difference (*p* < 0.01) between them. Among these four grassland types, the difference between the Pielou indices of WG plots showed the highest variation, resulting in an opposite trend (Figure 5d). Based on these results, it was implied that both TG and TS were more vulnerable to *A. adenophora* invasion than WG and WS.

### 3.6. Beta Diversity

The invasion and non-invasion plots of TG and TS exhibited moderate dissimilarity with Jaccard similarity indices of 0.2185 and 0.2012, respectively (Table 1). WG and WS also exhibited moderate dissimilarity with Jaccard similarity indices of 0.2698 each. This indicates that the invasion of *A. adenophora* remarkably altered grassland community assembly, as reflected by the greater differences in species composition and the reduction in the number of commonly shared species.

Sorenson similarity analysis showed moderate dissimilarity between the invasion and non-invasion plots of TG and TS, with similarity indices of 0.3587 and 0.3350, respectively (Table 2). There was moderate dissimilarity between WG and WS, with Sorenson’s similarity indices both being 0.4250. These values were consistent with Jaccard’s similarity indices, indicating that the invasion of *A. adenophora* resulted in a decrease in plant species diversity within the *A. adenophora*-invaded grassland community.

### 3.7. The Regression Analysis Between A. adenophora Importance Values (IV) and Grassland Flora Alpha Diversity

**Figure 6:**
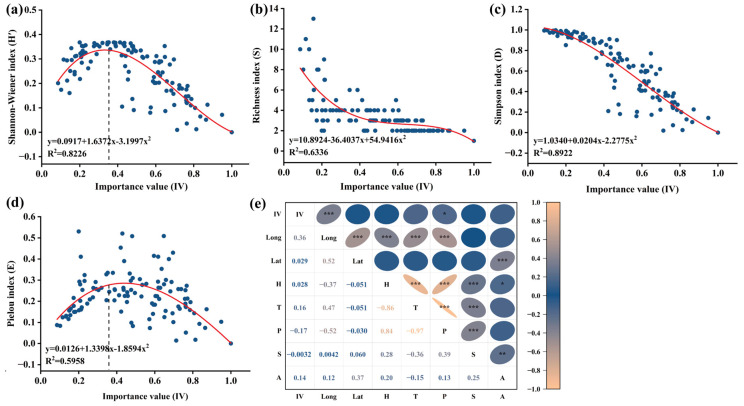
Regression analysis of the important *A. adenophora* value and Alpha diversity. (**a**) Shannon–Wiener index; (**b**) Richness index; (**c**) Simpson index; (**d**) Pielou index; (**e**) correlation analysis between important value and ecological indicators of *A. adenophora* distribution (* *p* ≤ 0.05, ** *p* ≤ 0.01, *** *p* ≤ 0.001; IV, important value of *A. adenophora*; Long, longitude; Lat, latitude; H, altitude; T, average annual temperature; P, annual precipitation; S, slope; A, slope aspect).

The regression analysis results showed that, within the invaded plots, there was a highly significant linear relationship between *A. adenophora* IVs and the Simpson index (R^2^ = 0.8922) (Figure 6c), followed by the Shannon–Wiener index (Figure 6a) (R^2^ = 0.8226), the Richness index (Figure 6b) (R^2^ = 0.6336), and the Pielou index (Figure 6d) (R^2^ = 0.5958). Specifically, it demonstrated that both the Shannon–Wiener index and Pielou index inverted U-shaped correlated with IVs of *A. adenophora*, and once IVs increase and reach the threshold 0.36, this would decrease the two indices (Figure 6a,d). These findings revealed that *A. adenophora* invasion exerted a sophisticated effect on grassland community dynamics, the IVs of *A. adenophora*, and the extent of invasion significantly shaped the flora composition assemblage.

Meanwhile, *A*. *adenophora’s* importance values were significantly positively correlated with longitude (*p* < 0.01). In contrast, they were significantly negatively correlated with annual precipitation, with a coefficient of 0.17 (*p* < 0.01) (Figure 6e). These results indicated that the colonization and invasion degree of *A. adenophora* were determined by multiple ecogeographical factors, including altitude, average annual temperature, and annual precipitation.

### 3.8. The Inhibitory Effect of A. adenophora Aqueous Extracts on Seedling Growth of Native Plants

**Figure 7:**
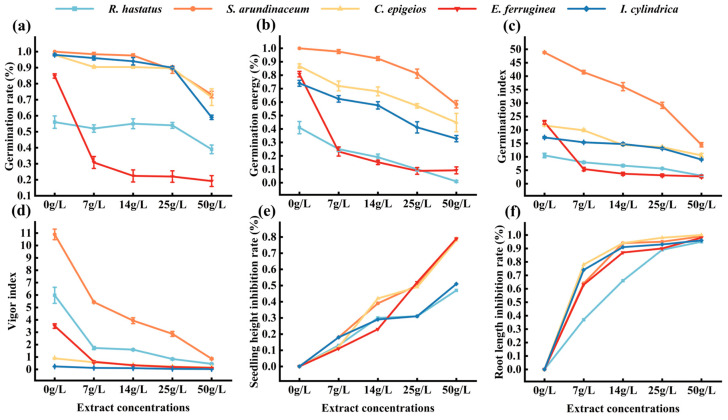
Allelopathic effect of *A. adenophora* leaf aqueous extract on the growth of native plants. (**a**) Germination rate; (**b**) germination energy; (**c**) germination index; (**d**) vigor index; (**e**) seedling height inhibition rate; (**f**) root length inhibition rate. Error bars represent mean ± standard error (n = 5).

For screening potential native plant species in grassland, five herbaceous species with economic value and ecological importance, *I. cylindrica*, *S. arundinaceum*, *E. ferruginea*, *C. epigeios*, and *R. hastatus*, identified in both invasion and no-invasion plots (Figure 4b,c), were selected for laboratory bioassay. The results showed that germination rates (Figure 7a), germination energy (Figure 7b), germination index (Figure 7c), and vigor index (Figure 7d) of the five plants decreased with increasing concentrations of *A. adenophora* leaf aqueous extract. Inhibition rate is a common indicator in plant physiology experiments, used to assess the impact of a treatment on plant growth by comparing growth differences between the treatment and control groups [39]. Specifically, the aqueous extract exerted an inhibitory effect on seedling height and root length of all tested plants (Figure 7e,f). However, *S. arundinaceum* exhibited the best integrative performance across five indices: germination rate, germination energy, germination index, vigor index, and seedling height inhibition rate. These results indicated that *S. arundinaceum* demonstrated the highest tolerance to *A. adenophora* leaf aqueous allelochemicals.

The seedling growth index of indigenous plant species under stress from *A. adenophora* leaf aqueous extract likely reflects these species’ richness and competitiveness within *A. adenophora*-invaded plots. Spearman correlation analysis was conducted between the seedling growth indices of the screened native plant species and corresponding field data to verify this hypothesis. Table 3 indicates that, except for *Rumex hastatus* and *Imperata cylindrica*, germination energy, vigor index, seedling height, and root length were correlated with relative density and height of *S. arundinaceum, C. epigeios*, and *E. ferruginea* (Table 3).

Combined with seedling growth indices (Figure 7; Table S2), it shows that *A. adenophora* leaf aqueous extracts imposed allelopathic detrimental effects on native plant seedling growth.

## 4. Discussions

### 4.1. The Impact of A. adenophora Invasion on the Grassland Plant Diversity

A considerable body of literature has reported that *A. adenophora* is capable of adapting to a diverse range of habitats, such as uncultivated land, cropland, woodland, and roadsides [17,18,40]. Generally, within three to five years of invasion, native herbaceous plants are directly or indirectly affected by competitive exclusion, leading to the exclusive community formation by *A. adenophora*. This process results in a decrease in the number and abundance of native species within the invaded habitats. In the present survey, all *A. adenophora*-invaded grassland plots exhibited a trend of decreasing plant species richness, indicating that the invasion has significantly altered the species composition and structure of the grassland ecosystem. Therefore, the declining or absent native plant species in the invaded plots were squeezed from the original grassland patches (Figure 4b,c). Alpha diversity analysis revealed that in *A. adenophora*-invaded plots, the Pielou index, Richness index, Shannon–Wiener index, and Simpson index were significantly lower than those of the non-invaded plots across different grassland types. Similar studies have also demonstrated that plant species index decreased significantly or very significantly in habitats severely invaded by *A. adenophora* [41,42]. Under conditions of abundant ecological niches in specific ecosystems, *A. adenophora* invasion can facilitate the colonization of indigenous plant species [43]. Similarly, in the present investigation, some indigenous plant species such as *D. viscosa*, *P. discolor*, and *C. megalanthum* were found in invasion plots but were absent in non-invaded ones (Figure 4b,c).

Beta diversity indices are defined to assess the degree of change in species composition within biocoenosis across environmental gradients, reflecting not only the distribution of species diversity within a region but also the relationship between species and their environment [44]. In the present research, Beta diversity analysis of plants showed that the Jaccard similarity index and Sorenson similarity index exhibited moderate dissimilarity between the invasion and non-invasion plots of TG and TS, whereas they exhibited high dissimilarity in WG and WS. The differences in beta diversity among different grassland types may be attributed to the inherent productivity, soil properties, and functional traits of native species pools [7,9]. TG and TS have higher productivity and more herbaceous species with weak competitive ability, making them more susceptible to invasion and thus greater changes in community composition; WG and WS have lower productivity and more shrub and perennial grass species with strong competitive ability, resulting in smaller changes in community composition after invasion. In conclusion, the dissimilarity of Beta diversity across different grassland types is probably associated with the sophisticated invasion process of *A. adenophora* and the specific species composition of each grassland type. In addition, low species similarity within an ecosystem indicates high variation in species composition, a low number of shared species, and high variability in species turnover [45]. The present findings provide complementary evidence that *A. adenophora* possesses remarkable adaptive, competitive, and invasive abilities compared to co-occurring plant species in grasslands of differing ecosystem types and species richness, potentially resulting in varied consequences depending on the invaded grassland type.

### 4.2. The Relationship Between A. adenophora Importance Values and Flora Community Diversity

The investigation of invasion plots showed that the importance values of *A. adenophora* range from 0.09 to 1.00 across all grassland types. Nonlinear curve-fitting regression analysis indicated that as the importance value of *A. adenophora* increased and reached the threshold of 0.36, the Shannon–Wiener and Pielou indices peaked, after which the alpha diversity index gradually declined (Figure 6a,d). This finding implied that, below an IV threshold of 0.36, an *A. adenophora* community with a lower dominance can contribute to the maintenance of indigenous flora diversity. This result aligns with previous studies reporting that *A. adenophora* invasion can, in certain contexts, lead to an increase in species richness [19,20]. Such increases may be attributed to niche complementarity between the invader and native species, or to the relatively weak competitive ability of certain species in uninvaded plots where competition among native species is high. In invaded communities, weak competitive pressure from *A. adenophora*, coupled with moderate levels of invasion-induced disturbance, may create new ecological niches for opportunistic native species, thereby promoting a slight increase in local species richness. This pattern is consistent with the intermediate disturbance hypothesis and echoes previous findings that biological invasions usually decrease biodiversity on a global scale but increase species diversity on a regional scale [46,47]. Although substantial observations have been documented in numerous contexts, the mechanisms underlying these phenomena are worth investigating through interspecific allelopathic assays between endangered grassland plant species and *A. adenophora.*

### 4.3. The Allelopathic Effect of A. adenophora on the Native Plants

The aggressive competitiveness of *A. adenophora* is attributed to its secretion of allelochemicals that suppress adjacent plant species in the ecosystem. It revealed that volatile compounds in its root secretions negatively affect seed germination in other plants [30]. Previous research reported that the aqueous extract of *A. adenophora* leaves inhibited *Triticum aestivum*, *Lens culinaris*, *Pinus roxburghii*, *Brassica campestris*, and *Quercus leucotrichophora* in terms of seed germination and seedling development efficiency [31,48]. However, the concentration gradients of aqueous extracts (50, 25, 14 g/L) used in the present study and in previous research appear to be higher than the natural allelochemical concentration in the soil, which is a validated method in allelopathic bioassays to amplify the inhibitory effect and screen plant species with strong tolerance [30,31]. Despite the inhibitory potential of *A. adenophora*, in the present study, both invasive and non-invasive plots commonly shared a certain number of plant species (Figure 4b,c). Therefore, it was rationally hypothesized that these coexisting indigenous plants can be more tolerant to the hydrosoluble allelochemicals in *A. adenophora* leaf tissue. One of the recipient native plant species, *S. arundinaceum*, showed strong tolerance against *A. adenophora* stress. A correlation analysis revealed a putative relationship between the seedling growth indices obtained from the bioassay and their growth indices (relative density, relative height, and relative coverage), indicating that the indicators of seed germination and seedling growth of native plants indirectly reflect the in situ population conditions (relative height, relative cover, and others) in grasslands. Therefore, it is rationally speculated that the use of *A. adenophora*-indigenous plant allelopathic bioassay results can estimate individual native plant species communities in situ within an *A. adenophora*-invaded ecosystem. However, more intensive research is required to provide supporting evidence.

Using indigenous plants with economic or ecological value in replacement control approaches has been validated as a promising alternative for IAP management. For example, *Sonneratia apetala* and *S. caseolaris* have been utilized to control *Spartina alterniflora* through alternative planting while promoting the restoration of native mangrove forests [49]. In the present study, the recipient plant *S. arundinaceum*, characterized by conspicuous tillering potential, multipurpose use, strong tolerance to diverse eco-geographical conditions, and genetic relation to sugarcane, combined with its superior tolerance to *A. adenophora* leaf aqueous extracts, makes it the optimal candidate for replacement control of *A. adenophora*. However, the feasibility of *S. arundinaceum* for replacement management was inferred solely from the laboratory bioassays and field investigation data analysis. It may pose potential risks if *S. arundinaceum* is introduced into non-native regions, for example, weedy behavior. Therefore, long-term localized field experiments remain necessary for further validation, and potential risks must also be evaluated prior to implementation.

## Figures and Tables

**Table 1:** Effects of *A. adenophora* invasion on the Jaccard similarity index of the grassland community.

	TG-I	TG-N	TS-I	TS-N	WG-I	WG-N	WS-I	WS-N
TG-I	—	0.2185	0.1919	0.1533	0.1558	0.1548	0.1370	0.1930
TG-N		—	0.1757	0.2066	0.1271	0.1417	0.1140	0.1176
TS-I			—	0.2012	0.1558	0.1548	0.1370	0.1930
TS-N				—	0.0820	0.1128	0.1200	0.1364
WG-I					—	0.2698	0.1200	0.1296
WG-N						—	0.1228	0.1148
WS-I							—	0.2698
WS-N								—

Note: Tropical grasslands (TG); tropical shrub-grasslands (TS); warm-temperate grasslands (WG); warm-temperate shrub-grasslands (WS);invasive plots (I); non-invasive plots (N); the same as below.

**Table 2:** Sorenson similarity index of the pairwise plots of the grassland community.

	TG-I	TG-N	TS-I	TS-N	WG-I	WG-N	WS-I	WS-N
TG-I	—	0.3587	0.3220	0.2659	0.2697	0.2680	0.2410	0.3235
TG-N		—	0.2989	0.3424	0.2256	0.2483	0.2047	0.2105
TS-I			—	0.3350	0.2697	0.2680	0.2410	0.3235
TS-N				—	0.1515	0.2027	0.2143	0.2400
WG-I					—	0.4250	0.2143	0.2295
WG-N						—	0.2188	0.2059
WS-I							—	0.4250
WS-N								—

**Table 3:** Correlation analysis between native plant seedling growth indices and investigation indicators in invasion plots.

Tested Plants	Field Indicators	Bioassay Indices					
		GR	GE	GI	VI	SH	RL
*Rumex hastatus*	RD	−0.06	0.11	0.10	0.22	0.20	0.11
	RH	0.06	0.08	0.14	0.06	0.08	0.10
	RC	0.16	0.17	0.19	0.13	0.16	0.17
*Saccharum arundinaceum*	RD	0.28	0.37 *	0.27	0.31 *	0.53 **	0.33 *
	RH	−0.26	−0.30 *	−0.25	−0.29	−0.35 *	−0.28
	RC	0.27	0.17	0.24	0.23	0.07	0.18
*Calamagrostis epigeios*	RD	−0.27	−0.19	−0.34 *	−0.32 *	−0.23	−0.40 **
	RH	0.05	−0.07	−0.21	−0.08	−0.18	−0.20
	RC	0.21	0.34	0.48	0.36	0.37	0.48
*Eragrostis ferruginea*	RD	−0.23	−0.22	−0.27	−0.34 *	−0.50 **	−0.40 *
	RH	−0.09	−0.05	−0.08	−0.07	−0.10	−0.06
	RC	−0.05	0.04	−0.01	0.08	0.29	0.28
*Imperata cylindrica*	RD	−0.08	0.04	0.01	0.09	0.08	0.18
	RH	−0.12	0.05	−0.01	−0.15	−0.31	−0.06
	RC	−0.08	−0.19	−0.15	0.09	0.08	0.08

GR, germination rate; GE, germination energy; GI, germination index; VI, vigor index; SH, seedling height; RL, root length; RD, relative density; RH, relative height; RC, relative coverage. * indicates significant difference (*p* < 0.05); ** exhibits highly significant difference (*p* < 0.01).

## Data Availability

All data generated and analyzed during this study are included in this published article and its Supplementary Information Files; further inquiries can be directed to the corresponding author. The software and codes used in this study were described in the Section 2.

## Supplementary Materials

The following supporting information can be downloaded at: https://www.mdpi.com/article/10.3390/plants15060862/s1, Table S1: Statistics of grassland types and acreage in Chengjiang County; Table S2: Effects of *A. adenophora* leaf extracts on seedling height and root length of native plants. Table S3: Species Lists of Plant Communities in invasion and non-invasion grasslands.
