# Supplementary material for: Landscape Heterogeneity Drives Plant Assemblage Dynamics and Invasibility of Semi-Natural Grasslands Under the Long-Term Invasion of Ageratina adenophora
Source: Plants (Basel). 2026 Mar 11;15(6):862. doi: 10.3390/plants15060862 (PMC13030750; doi:10.3390/plants15060862)
Supplement: Supplementary file 1 [file plants-15-00862-s001.zip › plants-4150051-supplementary.pdf]

Supplementary Table S1: Statistic of grasslands types and acreage in Chengjiang County

Towns	Number of grassland patches	Grasslands types and corresponding acreage (hm^2)				
		WG	WS	TG	TS	Sum
Fenglu	23	—	—	0.191	11.820	12.011
Longjie	673	110.240	17.242	663.871	509.311	1300.664
Yangzong	687	—	—	680.359	418.265	1098.624
Jiucun	445	—	—	80.591	357.464	438.055
Yousuo	287	—	—	131.775	298.867	430.642
Haikou	272	—	—	110.261	221.108	331.369
Sum	2387	110.240	17.242	1667.048	1816.835	3611.365

Note: The data retrieved from the 3rd National Land Resource Survey Report of China (Ministry of Natural Resources).

Supplementary Table S2: Effects of *A. adenophora* leaf extracts on seedling height and root length of native plants

Treatments		*R. hastatus*	*S.arundinaceum*	*C. epigeios*	*E. ferruginea*	*I. cylindrica*
**Seedling height (cm)**	0g/L	2.13±0.08a	2.90±0.06a	1.15±0.05a	2.29±0.06a	1.32±0.03a
	7g/L	1.86±0.10a	2.37±0.04b	1.01±0.03b	2.05±0.06b	1.08±0.02c
	14g/L	1.5±0.02b	1.78±0.12c	0.67±0.03d	1.77±0.06C	0.94±0.03d
	25g/L	1.48±0.09b	1.44±0.06d	0.59±0.03e	1.1±0.05e	0.91±0.02d
	50g/L	1.12±0.09c	0.62±0.02e	0.25±0.02f	0.47±0.02f	0.65±0.03e
**root length (cm)**	0g/L	9.27±0.25a	3.36±0.13a	3.68±0.19a	5.34±0.25a	1.53±0.08a
	7g/L	5.85±0.06c	1.20±0.05c	0.29±0.01cd	2.00±0.29b	0.39±0.02cd
	14g/L	3.17±0.10e	0.21±0.02e	0.24±0.03cde	0.7±0.05de	0.14±0.01f
	25g/L	1.05±0.13f	0.16±0.02e	0.07±0.01ef	0.52±0.03de	0.11±0.01f
	50g/L	0.46±0.06f	0.05±0.00e	0.00±0.00f	0.09±0.01f	0.06±0.01f

Different lowercase letters in the same row indicate significant differences among different extract concentrations for the same plant species ($p < 0.05$).

Supplementary Table S3: Species Lists of Plant Communities in invasion and non-invasion grasslands

Species	TG-I	TG-N	TS-I	TS-N	WG-I	WG-N	WS-I	WS-N
Artemisia argyi	1	1	1	1	1	1	1	1
Pteridium aquilinum	1	1	1	1	1	1	1	1
Bidens pilosa	1	1	1	1	1	1	—	—
Rumex hastatus	1	1	1	1	1	1	—	—
Rubus parvifolius	1	1	1	1	—	1	1	1
Imperata cylindrica	1	1	1	1	—	—	1	1
Origanum vulgare	1	1	1	1	—	1	—	—
Agrimonia pilosa	1	1	1	1	—	—	—	—
Digitaria sanguinalis	1	1	1	1	—	—	—	—
Equisetum ramosissimum	1	1	1	1	—	—	—	—
Eragrostis ferruginea	1	1	1	1	—	—	—	—
Pyracantha fortuneana	1	1	1	1	—	—	—	—
Saccharum arundinaceum	1	1	1	1	—	—	—	—
Buddleja officinalis	1	1	1	—	1	1	—	—
Datura stramonium	1	1	1	—	—	—	—	—
Ageratina adenophora	1	—	1	—	1	—	1	—
Clematis yunnanensis	1	—	1	—	—	—	—	—
Phytolacca acinosa	1	—	1	—	—	—	—	—
Thalictrum aquilegiifolium	1	1	—	1	1	1	—	—
Capillipedium parviflorum	1	1	—	1	—	1	1	1
Aegopodium podagraria	1	1	—	1	—	—	—	1
Dipsacus asper	1	1	—	1	—	—	—	1
Cirsium japonicum	1	1	—	1	—	—	—	—
Eleusine indica	1	1	—	1	—	—	—	—
Geranium sinense	1	1	—	1	—	—	—	—
Pseudognaphalium affine	1	1	—	1	—	—	—	—
Sida szechuensis	1	1	—	1	—	—	—	—
Coriaria nepalensis	1	1	—	—	1	1	1	1
Senecio scandens	1	1	—	—	1	1	—	—
Delphinium grandiflorum	1	1	—	—	—	—	—	—
Geum aleppicum	1	1	—	—	—	—	—	—
Nicandra physalodes	1	1	—	—	—	—	—	—
Smilax china	1	1	—	—	—	—	—	—
Tithonia diversifolia	1	1	—	—	—	—	—	—
Artemisia selengensis	1	—	—	—	—	—	—	—
Cynoglossum amabile	1	—	—	—	—	—	—	—
Erigeron canadensis	—	1	1	1	1	1	—	1
Calamagrostis epigeios	—	1	1	1	1	1	—	—
Trifolium repens	—	1	1	1	1	1	—	—
Elsholtzia rugulosa	—	1	1	1	—	—	1	1
Stellaria vestita	—	1	1	1	—	—	1	1
Duhaldea cappa	—	1	1	1	—	—	—	—
Leontopodium leontopodioides	—	1	1	1	—	—	—	—
Lotus corniculatus	—	1	1	1	—	—	—	—
Picris divaricata	—	1	1	1	—	—	—	—
Rubus rosifolius	—	1	1	1	—	—	—	—

Solanum aculeatissimum	—	1	1	1	—	—	—	—
Osteomeles schwerinae	—	—	1	1	1	1	1	1
Rubus ellipticus	—	—	1	1	1	1	1	1
Anaphalis margaritacea	—	—	1	1	—	—	1	1
Argentina lineata	—	—	1	1	—	—	—	1
Commelina communis	—	—	1	1	—	—	—	—
Isodon eriocalyx	—	—	1	1	—	—	—	—
Medicago sativa	—	—	1	1	—	—	—	—
Mentha canadensis	—	—	1	1	—	—	—	—
Scirpus rosthornii	—	—	1	1	—	—	—	—
Ipomoea purpurea	—	—	1	—	1	—	—	—
Dodonaea viscosa	—	—	1	—	—	1	1	—
Equisetum arvense	—	—	1	—	—	—	—	—
Urtica fissa	—	—	1	—	—	—	—	—
Cyperus cyperoides	—	1	—	1	—	—	1	1
Hypericum augustinii	—	1	—	1	—	—	1	1
Artemisia japonica	—	1	—	1	—	—	—	—
Berberis julianae	—	1	—	1	—	—	—	—
Cymbopogon goeringii	—	1	—	1	—	—	—	—
Ficus tikoua	—	1	—	1	—	—	—	—
Hypoestes triflora	—	1	—	1	—	—	—	—
Justicia procumbens	—	1	—	1	—	—	—	—
Persicaria capitata	—	1	—	1	—	—	—	—
Pogonatherum paniceum	—	1	—	1	—	—	—	—
Sida rhombifolia	—	1	—	1	—	—	—	—
Urena lobata	—	1	—	1	—	—	—	—
Verbena officinalis	—	1	—	1	—	—	—	—
Apluda mutica	—	1	—	—	1	1	—	—
Saccharum rufipilum	—	1	—	—	1	1	—	—
Solanum americanum	—	1	—	—	1	1	—	—
Phragmites australis	—	1	—	—	1	—	—	—
Carex baccans	—	1	—	—	—	—	1	1
Cyanotis arachnoidea	—	1	—	—	—	—	1	1
Clinopodium megalanthum	—	1	—	—	—	—	1	—
Lactuca tatarica	—	1	—	—	—	1	—	—
Berberis amurensis	—	1	—	—	—	—	—	—
Carpesium cernuum	—	1	—	—	—	—	—	—
Fagopyrum dibotrys	—	1	—	—	—	—	—	—
Galinsoga quadriradiata	—	1	—	—	—	—	—	—
Galium spurium	—	1	—	—	—	—	—	—
Isodon excisus	—	1	—	—	—	—	—	—
Lactuca indica	—	1	—	—	—	—	—	—
Myrsine africana	—	1	—	—	—	—	—	—
Odontosoria chinensis	—	1	—	—	—	—	—	—
Oenothera biennis	—	1	—	—	—	—	—	—
Prinsepia utilis	—	1	—	—	—	—	—	—
Ricinus communis	—	1	—	—	—	—	—	—
Salvia japonica	—	1	—	—	—	—	—	—
Sambucus javanica	—	1	—	—	—	—	—	—

Saussurea japonica	—	1	—	—	—	—	—	—
Sonchus oleraceus	—	1	—	—	—	—	—	—
Sporobolus fertilis	—	1	—	—	—	—	—	—
Arundinella setosa	—	—	—	1	1	1	—	—
Juncus effusus	—	—	—	1	—	—	1	1
Microstegium vimineum	—	—	—	1	—	—	1	1
Potentilla discolor	—	—	—	1	—	—	1	—
Lespedeza bicolor	—	—	—	1	—	1	—	—
Eragrostis pilosa	—	—	—	1	—	—	—	1
Angelica archangelica	—	—	—	1	—	—	—	—
Arthraxon hispidus	—	—	—	1	—	—	—	—
Axonopus compressus	—	—	—	1	—	—	—	—
Boehmeria nivea	—	—	—	1	—	—	—	—
Clinopodium chinense	—	—	—	1	—	—	—	—
Crotalaria ferruginea	—	—	—	1	—	—	—	—
Cyperus rotundus	—	—	—	1	—	—	—	—
Dysphania ambrosioides	—	—	—	1	—	—	—	—
Epilobium hirsutum	—	—	—	1	—	—	—	—
Euphorbia esula	—	—	—	1	—	—	—	—
Euphorbia fischeriana	—	—	—	1	—	—	—	—
Euphorbia jolkinii	—	—	—	1	—	—	—	—
Fagopyrum tataricum	—	—	—	1	—	—	—	—
Gonostegia hirta	—	—	—	1	—	—	—	—
Impatiens uliginosa	—	—	—	1	—	—	—	—
Laggera alata	—	—	—	1	—	—	—	—
Lespedeza cuneata	—	—	—	1	—	—	—	—
Mimosa pudica	—	—	—	1	—	—	—	—
Miscanthus sinensis	—	—	—	1	—	—	—	—
Ophiopogon bodinieri	—	—	—	1	—	—	—	—
Pedicularis sylvatica	—	—	—	1	—	—	—	—
Pennisetum flaccidum	—	—	—	1	—	—	—	—
Poa annua	—	—	—	1	—	—	—	—
Rubus idaeus	—	—	—	1	—	—	—	—
Setaria palmifolia	—	—	—	1	—	—	—	—
Tagetes erecta	—	—	—	1	—	—	—	—
Torilis japonica	—	—	—	1	—	—	—	—
Vaccinium fragile	—	—	—	1	—	—	—	—
Bothriochloa ischaemum	—	—	—	—	1	1	—	1
Amphicarpaea edgeworthii	—	—	—	—	1	1	—	—
Debregeasia orientalis	—	—	—	—	1	1	—	—
Cynoglossum furcatum	—	—	—	—	—	1	—	—
Malvastrum coromandelianum	—	—	—	—	—	1	—	—
Pennisetum alopecuroides	—	—	—	—	—	1	—	—
Gentiana cruciata	—	—	—	—	—	—	—	1
Pistacia weinmanniifolia	—	—	—	—	—	—	—	1

Dash (—) indicates species not recorded, and 1 indicates species recorded in the grassland type; TG-I: tropical grasslands invasion plots; TG-N: tropical grasslands non-invasion plots; TS-I: tropical shrub-grasslands invasion plots; TS-N: tropical shrub-grasslands non-invasion plots; WG-I: warm-temperate grasslands invasion plots; WG-N: warm-temperate grasslands non-invasion plots; WS-I: warm-temperate

shrub-grasslands invasion plots; WS-N: warm-temperate shrub-grasslands non-invasion plots.
